# The safety of BCG vaccination in cattle: results from good laboratory practice safety studies in calves and lactating cows

**DOI:** 10.1016/j.heliyon.2022.e12356

**Published:** 2022-12-15

**Authors:** Gareth A. Williams, Emer Scott-Baird, Alejandro Núñez, Francisco J. Salguero, Emma Wood, Steve Houghton, H. Martin Vordermeier

**Affiliations:** a Animal and Plant Health Agency (APHA), Addlestone, Surrey, KT15 3NB, UK; b Drayton Animal Health, Stratford-upon-Avon, Warks, CV37 9RQ, UK; c United Kingdom Health Security Agency (UKHSA), Salisbury, Wilts, SP4 0JG, UK; d Queens Hall, Narberth, Pembs, SA67 7AS, UK; e Veterinary Vaccines Consultancy Ltd, Paulerspury, Northants, NN12 7NN, UK

**Keywords:** Bovine tuberculosis, BCG vaccine, Safety data, Regulatory studies, Good laboratory practice

## Abstract

Bovine tuberculosis (bTB) is a global disease of livestock that has damaging economic, animal health and public health consequences. Conventional bTB disease control strategies, based around the testing and slaughter of cattle infected with bTB, are typically used to help limit or reduce the transmission of this disease but in many low- and middle-income countries such strategies may often be economically unviable, culturally unacceptable or logistically impracticable. The use of vaccination to protect cattle against bTB could provide a potentially more affordable, ethically acceptable and practical additional disease control measure. The protective efficacy of the commercially produced and readily available human vaccine against tuberculosis (*Mycobacterium bovis* Bacille Calmette-Guérin; BCG) in cattle has been demonstrated in many experimental laboratory and field studies. However, Good Laboratory Practice (GLP) studies assessing the safety of BCG vaccination in cattle have not previously been reported. We describe here the results of two GLP safety studies in which calves and lactating cows were vaccinated with BCG (Danish 1331 strain). From an animal health and welfare perspective, the results of these studies indicate that BCG vaccine is well tolerated in these categories of cattle with only transient and minor local or systemic reactions. Furthermore, there was no evidence that BCG was shed in raw milk, saliva or faeces collected from vaccinates and vaccination did not have a detrimental effect on milk yields in lactating cattle. These data, underpinned by GLP principles, further support the existing data on the safety of BCG vaccine in cattle and complement the abundant available cattle efficacy data for this potential cattle bTB vaccine.

## 1 Introduction

*Mycobacterium bovis* is the most common causative agent of bovine tuberculosis (bTB) disease in cattle and continues to represent a zoonotic tuberculosis (zTB) health risk to humans, particularly in lower-income countries with high levels of bTB cattle endemicity [1, 2, 3, 4]. It has been estimated that there were 140,000 new active human zTB cases and 11,400 human zTB deaths globally in 2020 but also that limited tuberculosis diagnostic capability, disease health surveillance and disease reporting in lower-income countries likely mean that these estimates are conservative [5].

The World Health Organization (WHO), The World Organisation for Animal Health (WOAH, formerly OIE), The Food and Agricultural Organization of the United Nations (FAO) and The International Union Against Tuberculosis and Lung Disease (The Union) acknowledge the critical importance of gaining a better understanding of the current levels of zTB and of the need for a concerted global effort to reduce its transmission from animals to people [6]. Available effective tuberculosis vaccines for cattle and other livestock could contribute to a reduction in disease transmission between animals and humans and, in doing so, help to end zTB and ease the economic impact of the disease in cattle that continues to threaten livelihoods.

Regulations, strategies and programmes targeting bTB control in cattle have been in place in the UK for many decades to address the impact of this disease on the productivity and economic sustainability of the national cattle livestock industry and to reduce the public health risk [7, 8]. However, bTB infection in farmed cattle continues to be a recalcitrant problem despite considerable and sustained efforts on the part of government, farmers and other stakeholders to eradicate the disease [9, 10]. The transmission of *M. bovis* infection is complex and influenced by multiple and often interrelated risk factors that need to be addressed collectively to control or reduce bTB incidence and prevalence in cattle [11, 12, 13].

Numerous experimental and field studies carried out over the past century have demonstrated that vaccination of cattle with BCG (*Mycobacterium bovis* Bacille Calmette-Guérin) can lead to a reduction in *M. bovis* bacterial load and bTB disease pathology in infected animals [2, 14, 15, 16]. Direct and indirect efficacy effects of a safe and field-deployable cattle bTB vaccine based on BCG could play a future role in reducing disease prevalence and averting new incidences [17]. Any cattle BCG vaccination programme could be strategically targeted for maximum impact and used in conjunction with existing (and future novel) disease control tools, such as those that mitigate disease transmission between and within the national herd and the UK wildlife bTB disease reservoir maintenance host (the Eurasian badger, *Meles meles*) [15, 18, 19]. Global acceptance and support of the use of cattle BCG vaccination in the control of bTB will likely require disease control policy options that have been developed to reflect the local epidemiological situation and meet the disparate needs of stakeholders in different nations [20].

During the development of any veterinary vaccine, pivotal laboratory studies and ultimately wider field studies are required to assess the safety of the vaccine in the target species that support its proposed dose, administration route and usage [21, 22]. Such studies may also generate information in relation to the food chain (milk and beef products, for example), environmental and human safety, and any requirement for withdrawal periods (the time between veterinary medicine administration and animal slaughter/food production), but this paper primarily relates to the assessment of the health and welfare of vaccinated animals. We report here the results of two independent Good Laboratory Practice (GLP) safety studies in which calves and lactating cows were subcutaneously vaccinated with BCG Danish 1331. The aims of these studies were to collect robust data on any local or systemic reactions that occur and the overall health and welfare condition of animals following vaccination with BCG. Additionally, data were collected to evaluate the persistence of viable BCG in anatomical tissues of vaccinated animals and to assess possible excretion of viable BCG from vaccinates. The animal safety data collected in these GLP studies would be pertinent to any overall BCG Danish 1331 vaccine regulatory benefit-risk assessment needed before this cattle vaccine could be supplied and sold to help control bTB in farmed cattle.

## 2 Materials and methods

The studies were randomised single-dose BCG safety tests in specific categories of animals (calves and lactating cows) of the vaccine target species (cattle, *Bos taurus*). They were conducted in accordance with the principles of GLP as defined in Annex 1 Section II of Directive 2004/10/EC of the European Parliament and of the Council of 11 February 2004 on the harmonisation of laws, regulations and administrative provisions relating to the application of the principles of good laboratory practice and the verification of their applications for tests on chemical substances. Studies were designed to meet the requirements of European Pharmacopoeia (EP) Monograph 5.2.6: Evaluation of safety of veterinary vaccines and immunosera and VICH GL44 (2009): Target animal safety for veterinary live and inactivated vaccines [22]. These regulatory safety studies were not designed to test formal hypotheses in relation to treatment groups (vaccinates and controls). As such, the reporting of the data obtained is restricted to general descriptive statistics, supplemented with graphical, tabular or illustrative representations of these data, to avoid communicating any unwarranted or inappropriate statistical significance or inference.

### 2.1 Ethics statement

All animal work was carried out in accordance with the Animals (Scientific Procedures) Act 1986 and associated guidelines. Study designs were reviewed and approved by affiliate organisations’ internal ethical review panels (lactating cow study reference DAH AWERB PL40/3257; calf study reference APHA AWERB 70/7249-2-001) before the studies commenced and organisational codes of practice, working procedures and guidelines relating to animal care were followed throughout the studies. This paper was prepared with consideration to the ARRIVE Guidelines for reporting animal research [23].

### 2.2 Cattle

Animals used in the studies were either imported to the UK from Denmark (Officially Bovine Tuberculosis Free status as then defined by European Commission Decision 2003/467/EC) or were born to the imported animals in the UK and subsequently housed away from any potential sources of bTB infection, in UK Home Office-compliant accommodation. All animals that were imported from Denmark were confirmed to be bTB tuberculin skin test negative (single intradermal bovine tuberculin test) shortly before shipment to the UK and an average of 64 days before vaccination with BCG (range 53–71 days, SD 5). Appropriate biosecurity measures were taken to ensure there was no direct (or indirect, fomite-mediated) contact between vaccinate and control treatment group animals in the studies. A total of 67 Holstein or Holstein x Danish Red calves (33 females and 34 males, aged 26–57 days at the time of vaccination) completed the calf study and 40 lactating Holstein or Danish Red cows (female, aged 22–33 months at the time of vaccination, 4–8 days after calving) completed the lactating cow study. All animals were assessed to be clinically healthy by examining veterinary surgeons before inclusion in the studies.

### 2.3 Vaccine dose and vaccination

Lyophilised BCG Danish 1331 (BCG Vaccine SSI, Statens Serum Institute, Denmark at the time of the study; currently BCG Vaccine AJV, AJ Vaccines, Denmark), produced to Good Manufacturing Practice (GMP) standards, was reconstituted in a volume of the manufacturer's diluent sufficient to achieve a target range of 0.4–1.6 × 10^7^ CFU per single 0.5 ml dose based on the manufacturer's vial dose specifications (2–8 × 10^6^ CFU/vial) and administered to vaccination treatment groups within four hours of reconstitution. This dose was selected for the safety studies to ensure that all animals received a dose of at least 4 × 10^6^ CFU as this represents the maximum of the dose range of 1.0–4.0 × 10^6^ CFU; vaccination doses in this range have previously been demonstrated to provide protective efficacy against bTB disease [2, 15]. Control animals in each study were placebo-vaccinated using 0.5 ml of vaccine diluent.

Animals were randomised to either vaccine or control treatment groups in each study. In the calf study there were a total of 61 vaccinates and 6 control animals and in the lactating cow study there were a total of 20 vaccinates and 20 control animals. Approximately one week before vaccination, an area of approximately 10 cm^2^ in the middle third of the left side of each animal's neck was shaved to provide a clean and accessible area for vaccination (not near to any previous bTB tuberculin skin test sites). A similarly sized area was also prepared in approximately the same position on the right hand side of each animal's neck to subsequently serve as an uninjected comparator site to assess heat, pain and sensitivity at the site of injection. 0.5 ml of reconstituted BCG vaccine (0.4–1.6 × 10^7^ CFU dose) or 0.5 ml of control diluent were administered to vaccine and control treatment group animals respectively. Vaccine and placebo-vaccination vaccine diluent were administered by subcutaneous injection (21G × 25.4 mm/1″ needle) into the pre-prepared shaved areas on the left-hand side of the neck, avoiding the spinous processes and any visible or palpable pre-existing swellings or marks.

### 2.4 Cattle health and welfare observations

Animals were assessed for any immediate gross adverse effects of treatment by visual inspection (animal behaviour and physical appearance) immediately after vaccination and then at one hour after vaccination. Rectal body temperatures, physical appearance and the general behaviour of each animal (coughing/breathing, demeanour and food/water intake levels, for example) were monitored periodically throughout the duration of the studies by animal husbandry staff or attending veterinary surgeons.

### 2.5 Injection site examination for adverse reactions and cattle discomfort

Injection sites were examined by visual inspection and palpation to identify and monitor the size and appearance of any local reactions following vaccination (e.g. skin discolouration, broken skin and suppurative exudation). Additionally, heat, pain or sensitivity were assessed by comparing the temperature (hand tested) and the animal's physical reaction (e.g. twitching, movement, attempting to bite the injection site etc) during palpation of the site of injection to those observed during palpation of the uninjected comparator site. Skin thickness measurements (pinch fold) at the injection sites were also recorded using calibrated constant pressure skinfold callipers.

### 2.6 Raw milk: Yields and BCG culture

Lactating cows were milked twice daily using an abreast milking parlour from the time of calving to the end of the study and the daily milk yield (kg) recorded. Representative 25 ml samples of raw milk collected daily from each vaccinated lactating cow were submitted for BCG culture (estimated limit of detection: 186 CFU/raw milk sample) throughout the course of the study. In summary, 25 ml raw milk samples were separated into ‘cream’ and ‘cellular’ phases by centrifugation (10 min at 1100 × *g*) and each phase treated with 0.56 M ethanedioic acid (oxalic acid) to reduce or eliminate any non-mycobacterial contaminating organisms present. The two resulting decontaminated raw milk sample phases were each washed with 0.145 M sodium chloride to remove residual ethanedioic acid and finally resuspended in the same. Ten 0.2 ml aliquots of each decontaminated and washed milk sample phase were inoculated onto modified Middlebrook 7H11 agar plates and any BCG colonies cultured were enumerated after a total of 12 weeks' incubation at 37 °C. Raw milk samples collected from control lactating cows were not submitted for BCG culture.

### 2.7 BCG culture from faeces and saliva samples

Saliva swabs and faecal samples were collected from vaccinated calves at intervals throughout the course of the study and submitted for BCG culture (the estimated limits of detection were 341 CFU/faecal sample and 25 CFU/saliva sample). Following collection, each saliva swab sample was immersed in 3.5 ml Middlebrook 7H9 broth and briefly vortex mixed. Collected faecal samples were agitated with glass beads (∼5 mm diameter) in 0.145 M sodium chloride (using a volume in ml approximately equal to the individual faecal sample weight in g) to form a slurry and larger particulate matter present allowed to sediment overnight at 4–8 °C. 15 ml of the clearer upper faecal slurry phase was then treated with 0.56 M ethanedioic acid, washed with 0.145 M sodium chloride (10 mins at 1100 × *g*) and resuspended in the same. For each sample collected, two 0.2 ml processed saliva swab aliquots and two 0.2 ml processed faecal sample aliquots were each inoculated onto modified Middlebrook 7H11 agar and BCG colonies enumerated after a total of 12 weeks’ incubation at 37 °C. Saliva and faecal samples were not collected from control calves.

### 2.8 Post-mortem examination of cattle and BCG culture from tissue samples

**Figure 1:**
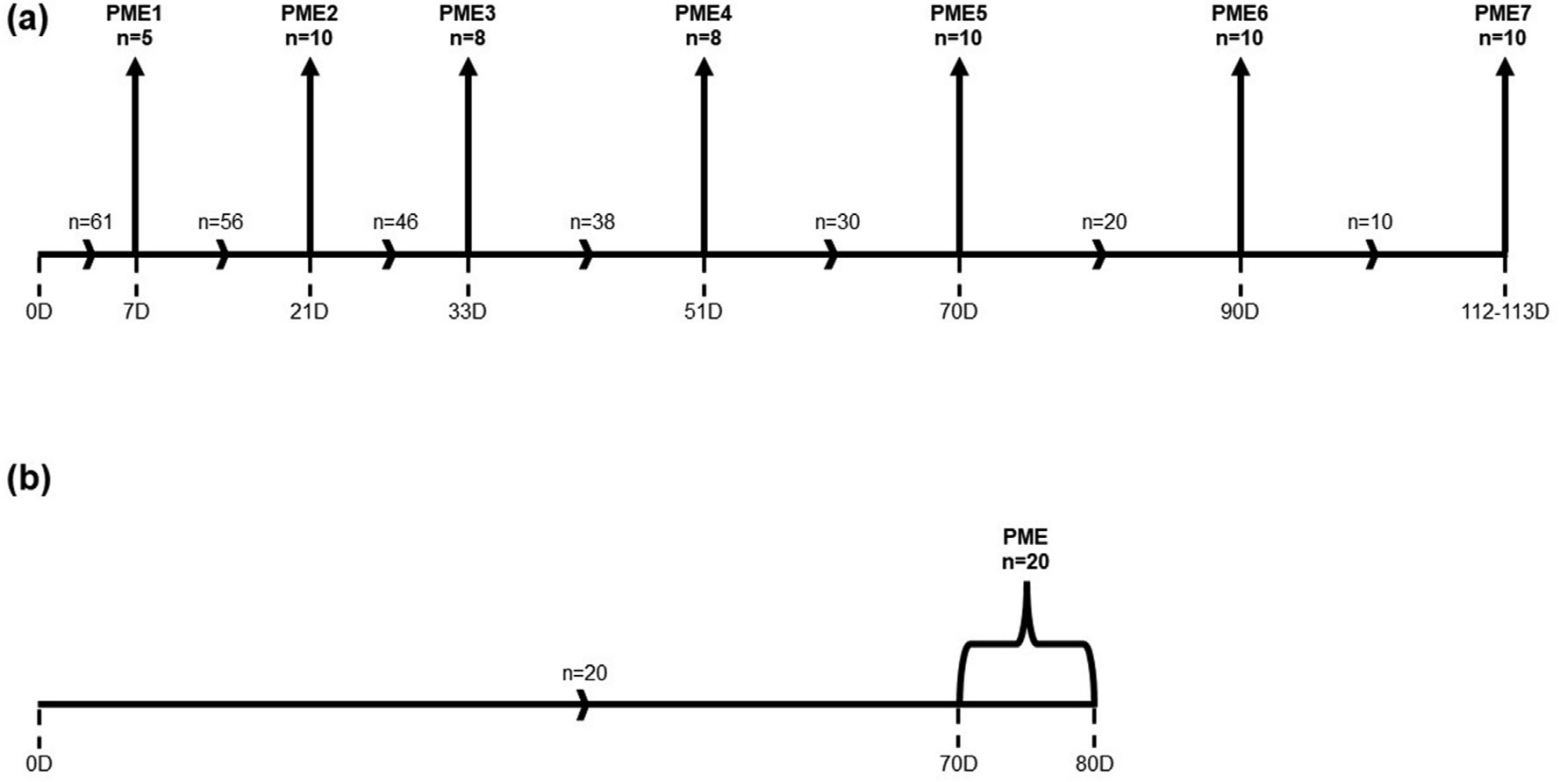
A schematic representation of the timeline of the GLP safety studies in BCG-vaccinated cattle. (a) Sixty-one calves were vaccinated with BCG at day zero (D0) and then examined at post-mortem in groups at seven designated time points (PME1-7) occurring at 7, 21, 33, 51, 70, 91 and 112–113 days (n = 5, n = 10, n = 8, n = 8, n = 10, n = 10, n = 10; respectively) after vaccination. (b) Twenty lactating cows vaccinated with BCG were also examined at post-mortem 70–80 days (mean 78 days, SD 2) after vaccination. This represented a single study end point however for logistical reasons at the time it was necessary to carry out post-mortem examinations on these animals over a ten-day period (D70: n = 1, D76: n = 3, D77: n = 3, D78: n = 5, D79: n = 3, D80: n = 5).

A study timeline showing post-mortem examination time points is provided in Figure 1. Groups of BCG-vaccinated calves were assigned to seven designated time points for euthanasia and post-mortem examination. Post-mortem examination groups were as follows: 7 days post vaccination (3 female and 2 male); 21 days post vaccination (5 female and 5 male); 33 days post vaccination (4 female and 4 male); 51 days post vaccination (4 female and 4 male); 70 days post vaccination (5 female and 5 male calves); 91 days post vaccination (5 female and 5 male); 112/113 days post vaccination (5 female and 5 male). All lactating cows vaccinated with BCG were euthanised and examined at post-mortem at 70–80 days post vaccination (mean 78 days, SD 2). All animals were examined for any gross pathological abnormalities including the presence of any tissue lesions characteristic of mycobacterial infection or colonisation. Following the removal of extraneous fat, post-mortem samples of injection site tissue (skin and underlying muscle), kidney, liver, lung, testicles/ovaries and lymph nodes (prescapular, caudal mediastinal, bronchial, ileocecal/mesenteric, mandibular, lateral retropharyngeal, popliteal and inguinal) were submitted for BCG culture (estimated limit of detection: 335 CFU/tissue sample). In summary, tissue samples were homogenised (Stomacher® 80 microBiomaster Lab Blender, Seward Limited, Worthing, UK) in 0.145 M sodium chloride and two 0.2 ml aliquots of the resulting homogenate each inoculated directly onto modified Middlebrook 7H11 agar. BCG colonies were enumerated after a total of 12 weeks’ incubation at 37 °C.

## 3 Results

### 3.1 Body temperatures and general health observations

**Figure 2:**
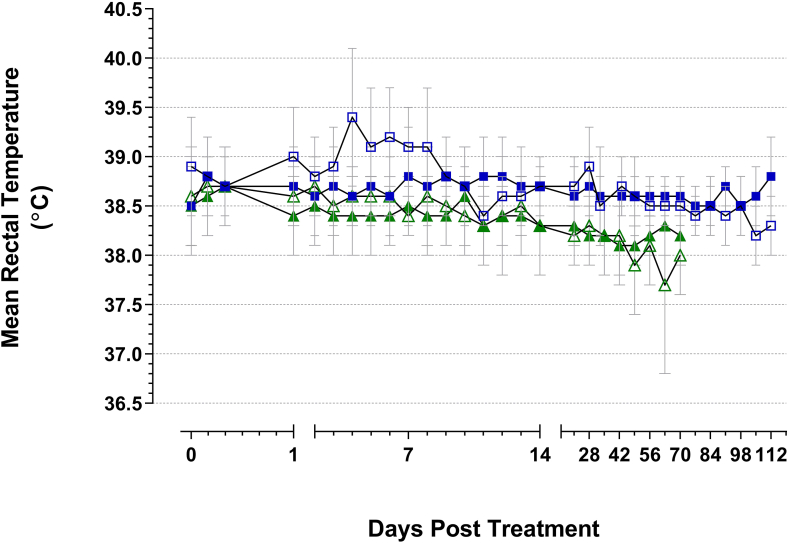
Mean rectal body temperatures (SD error bars) of animals in each treatment group measured throughout the course of the studies. Blue open squares: control calves; blue filled squares: BCG-vaccinated calves; green open triangles: control lactating cows; green filled triangles: BCG-vaccinated lactating cows.

The mean rectal temperature of calves and lactating cows immediately prior to treatment were 38.6 °C (SD 0.4, range 37.8–40.1 °C) and 38.5 °C (SD 0.5, range 37.5–39.8 °C), respectively. The maximum change in treatment group mean temperatures in the period 4–24 hours after vaccination with BCG or vaccine diluent were +0.1 °C 24 hours after treatment for control calves, +0.3 °C 4 hours after treatment for BCG-vaccinated calves, +0.2 °C 8 hours after treatment control lactating cows and +0.3 °C 8 hours after treatment for BCG-vaccinated cows. The maximum changes in individual animal temperatures observed during the same period were: +0.7 °C in 14% of control calves, +1.1 °C in 3% of BCG-vaccinated calves, -1.4 °C in 5% of control lactating cows and +1.3 °C in 5% of BCG-vaccinated lactating cows. Subsequent rectal temperatures fluctuated throughout the studies (Figure 2) but remained within the following ranges: control calves 38.0–40.4 °C (mean 38.7 °C, SD 0.4); BCG-vaccinated calves 37.9–40.9 °C (mean 38.7 °C, SD 0.4); control lactating cows 36.1–40.0 °C (mean 38.4 °C, SD 0.5); BCG-vaccinated calves 37.3–40.6 °C (mean 38.4 °C, SD 0.4).

As expected in any animal studies, veterinary intervention was required in some calves and lactating cows for health issues which were assessed to be unrelated to the vaccination treatment (e.g. pneumonia and conjunctivitis in calves; cuts, sores, injuries, mastitis, lameness, loose faeces, infections in lactating cows). In the first 14 days following vaccination, reduced dry compound feed consumption (inappetence) was observed in six of the BCG-vaccinated cows but the weights and overall health condition of the vaccinated group did not differ from those of the control group throughout the study. Aside from the transient inappetence in some vaccinated lactating cows, the general behaviour, physical health and feeding patterns of animals used in these studies was assessed to be normal by animal care staff.

### 3.2 Injection site examination

**Figure 3:**
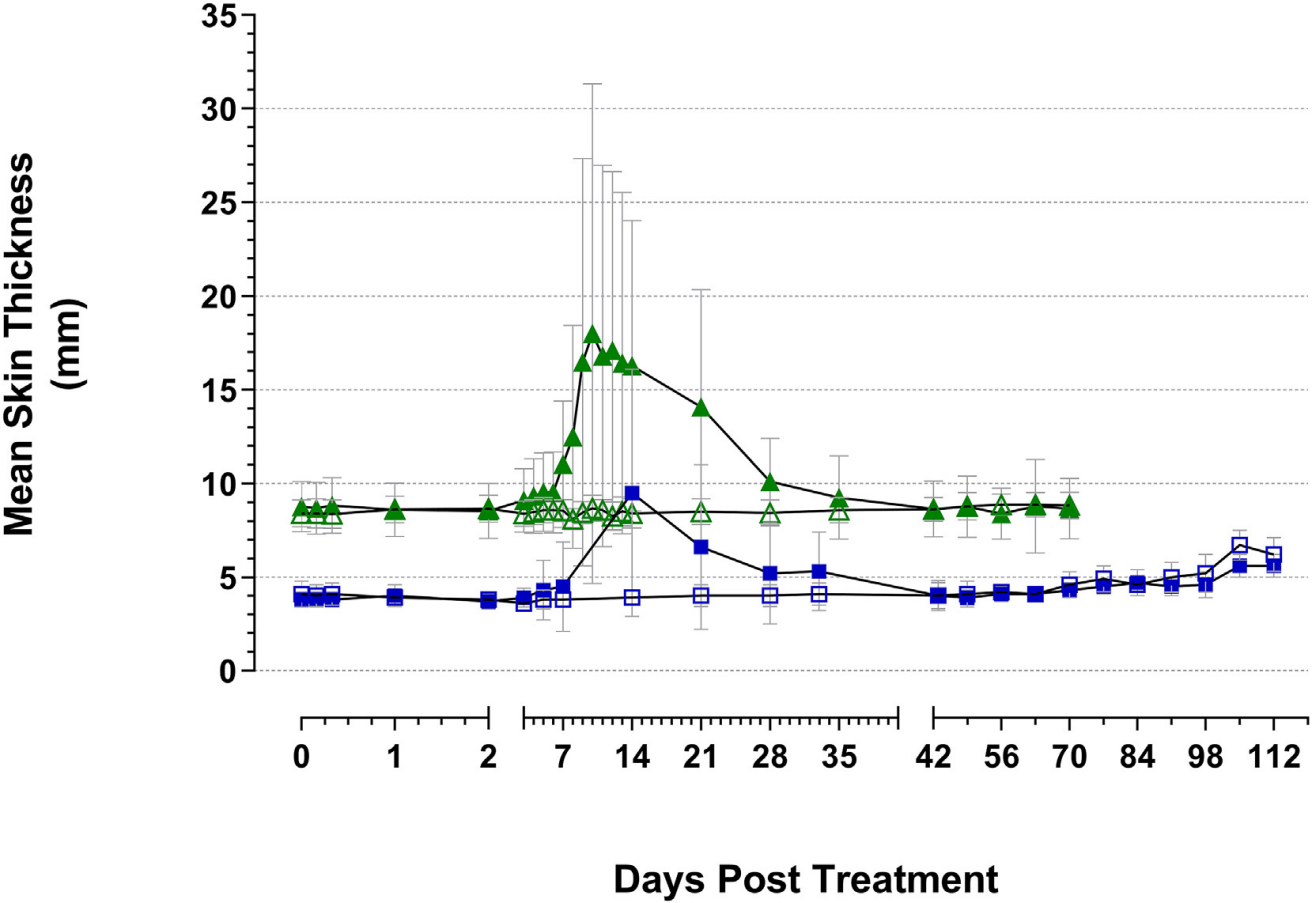
Mean injection site skin thicknesses (SD error bars) measured throughout the course of the studies: Blue open squares: control calves; blue filled squares: BCG-vaccinated calves; green open triangles: control lactating cows; green filled triangles: BCG-vaccinated lactating cows.

Immediately before treatment the mean skin thicknesses measured at the sites of injection were 3.8 mm (SD 0.4, range 3.0–5.2 mm) for the calves and 8.6 mm (SD 1.1, range 6.6–11.4 mm) for the lactating cows. The mean skin thicknesses at the injection site of both calves and lactating cows vaccinated with BCG began to increase approximately 3–5 days after treatment (Figure 3). The maximum increases in treatment group mean skin thicknesses, relative to the pre-treatment mean measurements, were recorded at 14 days after BCG vaccination of calves (+5.7 mm) and at 10 days after BCG vaccination of lactating cows (+9.2 mm). The thickness of the skin at BCG injection sites gradually decreased to values within the range of those obtained for each treatment group control animals in the period 5–6 weeks after treatment. Little or no increases in skin thicknesses were observed at the site of injection in control animals in each treatment group. During the studies, a discernible diffuse or circumscribed subcutaneous nodule could be palpated at the site of injection in 68% of calves and 88% of lactating cows vaccinated with BCG on at least one examination occasion after treatment. These nodules became predominantly more diffuse in nature as the studies progressed but continued to be detected at the site of injection in a proportion of BCG-vaccinated animals until the end of the study periods (30% of calves examined at 112 days after BCG vaccination and 20% of lactating cows examined at 70 days after BCG vaccination). There was no evidence of increased heat at the site of injection in either BCG-vaccinated lactating cows or BCG-vaccinated calves throughout the course of the studies; skin temperatures were qualitatively assessed to be the same temperature as the skin tissue surrounding the site of injection and as the uninjected comparator site skin. Similarly, there was no evidence of noteworthy increased pain or sensitivity at the site of injection; palpation of both uninjected comparator sites and injection sites of BCG-vaccinated calves and BCG-vaccinated lactating cows elicited either no discernible reaction in animals or only slight animal movement or twitching.

### 3.2 Milk yields of BCG-vaccinated lactating cows

**Figure 4:**
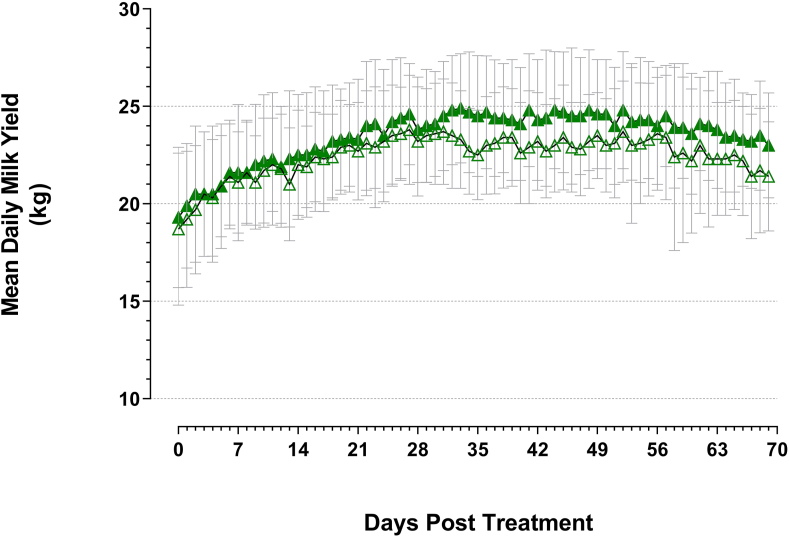
Mean milk yield (kg/day) of lactating cow treatment groups measured throughout the course of the study (SD error bars). Green open triangles: control lactating cows; green filled triangles: BCG-vaccinated lactating cows.

Mean daily milk yields of both control and BCG-vaccinated lactating cow treatment groups gradually increased following vaccination (coincidental with 4–8 days since calving), peaked at 33 days after control treatment (24.9 kg/day, SD 2.8) and 27 days after BCG treatment (23.8 kg/day, SD 2.8) and then gradually declined towards the end of the study (Figure 4). The mean daily milk yields of BCG-vaccinated lactating cows were equal to or marginally greater (mean +1.0 kg/day, SD 0.5) than control lactating cow daily milk yields at every sampling time point throughout the course of the study.

### 3.3 BCG excretion in saliva, faeces and raw milk samples in vaccinated animals

BCG was not cultured from any saliva or faecal samples collected (647 and 580 analysed in total, respectively) from BCG-vaccinated calves nor from any raw milk samples (520 analysed in total) collected from BCG-vaccinated lactating cows.

### 3.4 BCG culture at post-mortem examination

**Figure 5:**
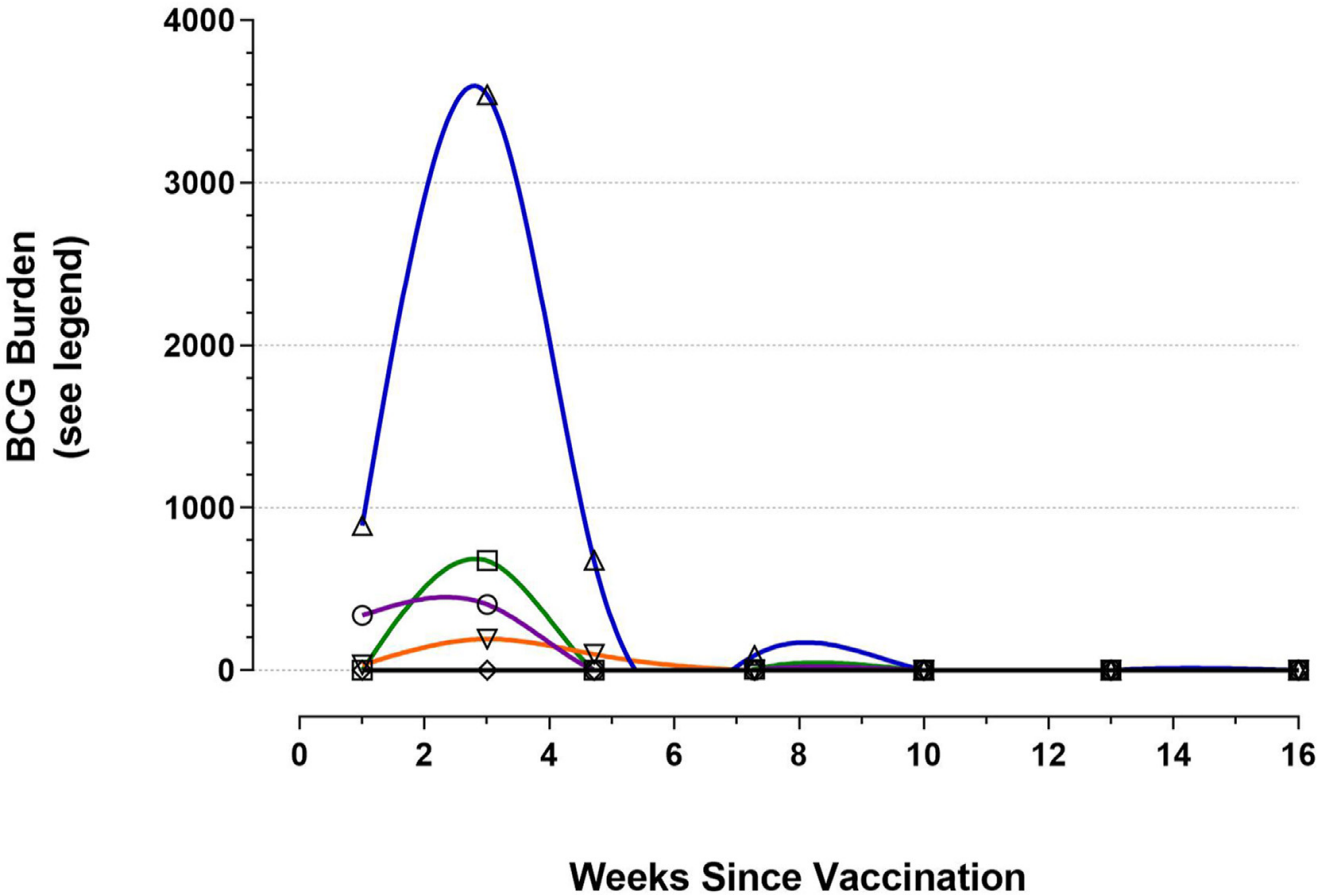
For illustrative purposes, a graphical representation of the BCG burden in calf tissues during the 16-week period following vaccination. Blue line/up triangle: left prescapular lymph node; green line/square: right prescapular lymph node; purple line/circle: injection site and underlying muscle; orange line/down triangle: caudal mediastinal-left bronchial lymph node pool; black line/diamond: lung. BCG burden values for each post-mortem examination time point were derived (normalised) by multiplying the mean BCG CFU/g of tissue sample values obtained by the proportion of calves in each post-mortem examination group that were BCG culture positive.

Tissue lesions characteristic of mycobacterial infection were not observed by examining pathologists in any BCG-vaccinated animals at post-mortem nor during any subsequent further histopathology investigations, where requested by pathologists. BCG was not cultured from post-mortem tissue samples of kidney, liver, testicles/ovaries nor from any ileocecal/mesenteric, mandibular, lateral retropharyngeal, popliteal and inguinal lymph node samples from BCG-vaccinated animals at any time during the duration of the studies. However, BCG was cultured at post-mortem from samples of injection site tissue, lung and a limited number of lymph nodes (left prescapular, right prescapular and caudal mediastinal-left bronchial pool) collected from BCG-vaccinated animals. The proportion of BCG culture-positive animals at each time point and the mycobacterial loads within post-mortem tissue sample were variable (Table 1). For illustrative purpose, Figure 5 provides a graphical representation of the post-mortem BCG tissue burden during the 16-week period following vaccination. BCG was not cultured from any calf post-mortem tissue samples collected 70 days after BCG vaccination but was cultured from the injection site tissue of 15% of BCG-vaccinated lactating cows at 78 days after vaccination.

**Table 1:** BCG culture from tissue samples collected from BCG-vaccinated calves and lactating cows. The proportions of animals from which BCG culture positive tissue samples were obtained are expressed as a fraction of those sampled at the time points given. The italicised values are the mean BCG CFU per gram of tissue sample in those animals that were BCG culture positive.

Animal Category (n)	Mean Days post BCG Vaccination	Proportion of animals sampled that were culture-positive *(mean BCG CFU/g sample in culture-positive vaccinates)*				
		Injection Site	Lung	Lymph Nodes		
				LPS^a^	CM-LB^b^	RPS^c^
Calves (5)	7	0.60 (*564)*	0.20 (*6)*	1.0 *(890)*	0.20 *(168)*	0
Calves (10)	21	0.40 *(1014)*	0	0.90 *(3935)*	0.20 *(966)*	0.10 *(6748)*
Calves (8)	33	0	0	0.75 *(916)*	0.38 *(261)*	0
Calves (8)	51	0	0	0.50 *(185)*	0	0.13 *(48)*
Calves (10)	70	0	0	0	0	0
Lactating Cows (20)	78	0.15 *(48)*	0	0	0	0
Calves (10)	91	0	0	0	0	0
Calves (10)	112	0	0	0	0	0

a left prescapular.

b caudal mediastinal-left bronchial pool.

c right prescapular.

## 4 Discussion

BCG has been used as an experimental tuberculosis vaccine in a wide range of animal species, including cattle, since the early 20th century with very few adverse reactions being reported [24]. This may be, in large part, because many of these studies were primarily designed to evaluate vaccine efficacy, explore immunological responses or assess other areas of scientific interest such as disease transmission. As such, these studies would not necessarily include a comprehensive and systematic assessment of the health and welfare status of BCG-vaccinated animals above and beyond that required as part of desirable or mandated good veterinary, animal welfare and ethical conduct and practices. Where there have been reports of potentially adverse reactions (such as the transient occurrence of vaccination site reactions, for example) in animals vaccinated with BCG, comparison of these with the results obtained in the studies reported here is difficult as the target species/breed/age, vaccine strains, vaccine doses and routes of administration have differed.

Despite the general absence of adverse BCG vaccination reactions in cattle having been reported in published scientific literature, data and information deficiencies remain with respect to the health and welfare of BCG-vaccinated cattle, at least from a necessarily vigorous regulatory perspective. The results presented here help to address some of these deficiencies. BCG vaccination appeared to be well tolerated with no indication of any significant pain, discomfort or distress in vaccinated cattle and generally unremarkable effects on the animal health and welfare parameters assessed. Transient and variable increases in body temperature, skin thickness/nodules at the site of injection and inappetence were observed in some animals but this did not negatively impact on their overall health condition which was assessed to be normal. BCG vaccination did not have a detrimental effect on milk yields in lactating animals, and indeed yields in BCG-vaccinated lactating cows were typically equal to or marginally greater than those of control lactating cows. This may indicate a possible nonspecific health and potential economic benefit of cattle BCG vaccination that has similarly been recently reported in a Chilean dairy farm herd cattle vaccinated with BCG (Russia strain) [25]. Such nonspecific (off-target) beneficial health effects have been reported in a range of animal species vaccinated with BCG and have included decreased severity of disease during parasitic infection, reduced pathogen multiplication during bacterial infections and even initiation of antitumor activity [26].

We have previously carried out two additional GLP safety studies in which calves (aged 15–32 days at first vaccination) and pregnant cows (in each trimester of pregnancy) were vaccinated subcutaneously with a tenfold overdose of BCG Danish 1331 followed by a single dose approximately seven weeks later (doses relative to the dose range in the studies reported here) [27, 28]. Similar transient mild local and systemic reactions were also observed in these BCG-vaccinated animals but, overall, attending veterinarians and animal welfare personnel assessed all vaccinates to have been generally healthy throughout these studies. Despite these animals receiving two separate successive BCG vaccinations, the first of which was approximately a tenfold greater dose than the studies reported here, the directors of these overdose studies concluded that BCG vaccination did not cause any events in animals that they considered to be adverse effects.

To maximise the data obtained from the animals used in the animal safety studies reported here, attempts were also made to culture viable BCG from faeces, saliva and raw milk samples collected from vaccinates while living and from tissue samples once euthanised. BCG was not cultured from the faeces, saliva or raw milk samples that were collected from vaccinated cattle but low levels (relative to the original vaccination dose) of viable BCG persisted and were cultured in the tissues of 63% of calves up to 51 days after vaccination and in 15% of lactating cows up to 78 days after vaccination. The persistence of viable BCG Danish in the tissues of vaccinates has previously been reported in other animal species (including food producing species) although the routes of vaccine administration, the duration for which this persistence is sustained, the proportion of vaccinated animals affected and the quantity of viable BCG cultured were variable [29, 30, 31, 32]. As cattle are a major food producing species, veterinary medicine regulatory authorities would need to consider the persistence of BCG in animal tissues in the context of any perceived food chain safety risk and in setting any veterinary medicine withdrawal periods for BCG-vaccinated livestock.

## 5 Conclusions

An abundance of available data and information, collected over the last century, indicates that the vaccination of cattle with BCG can be anticipated to be both safe from a cattle health and welfare perspective and able to provide a degree of protection against bTB disease. Additional BCG vaccination safety data, collected in larger numbers and different categories of cattle (other breeds and those pre-exposed to environmental mycobacterial species, for example) under UK field conditions will ultimately be required before a cattle BCG vaccine could become commercially available as a potential additional bTB disease control tool for the UK cattle livestock industry. The study results reported here further support the existing good safety profile of BCG vaccination in cattle and, atypically and beneficially, are underpinned by Good Laboratory Practice principles and the additional data quality and integrity assurances that these principles provide.

## Declarations

### Author contribution statement

Gareth Allen Williams: Wrote the paper.

Gareth Allen Williams Emer Scott-Baird; Alejandro Núñez; Francisco J Salguero; Emma J Wood: Performed the experiments; Analyzed and interpreted the data; Contributed reagents, materials, analysis tools or data.

Steve Houghton; H Martin Vordermeier: Conceived and designed the experiments.

### Funding statement

This work was supported by the UK Government’s Department for Environment, Food and Rural Affairs (Defra) as part of project code SE3266 (Continued vaccine development: Improving BCG and developing non-sensitising vaccines for cattle).

### Data availability statement

Data included in article/supp. material/referenced in article.

### Declaration of interest’s statement

The authors declare no competing interests.

### Additional information

© Crown copyright (2022).  Licensed under the Open Government Licence v3.0.
